# The Knowledge, Awareness, and Practices of Portuguese General Practitioners Regarding Multimorbidity and Its Management: Qualitative Perspectives from Open-Ended

**DOI:** 10.3390/ijerph13111097

**Published:** 2016-11-08

**Authors:** Filipe Prazeres, Luiz Santiago

**Affiliations:** 1 Faculdade de Ciências da Saúde, Universidade da Beira Interior, Covilhã 6200-506, Portugal; 2 Centro de Saúde de Aveiro, Aveiro 3810-000, Portugal; 3 Unidade de Saúde Familiar Topázio, Coimbra 3020-171, Portugal; lmsantiago@netcabo.pt

**Keywords:** Portugal, primary care, qualitative study, perceived experiences, multimorbidity

## Abstract

Multimorbidity’s high prevalence and negative impact has made it a subject of worldwide interest. The main aim of this study was to access the Portuguese knowledge, awareness, and practices of general practitioners (GPs) regarding multimorbidity and its management, in order to aid in the development of interventions for improving outcomes in multimorbid patients in primary care. A web-based qualitative descriptive study was carried out in the first trimester of 2016 with primary care physicians working in two districts of the Centre region of Portugal. Open-ended questions were analysed via inductive thematic content analysis. GPs pointed out several difficulties and challenges while managing multimorbidity. Extrinsic factors were associated with the healthcare system logistics’ management (consultation time, organization of care teams, clinical information) and society (media pressure, social/family support). Intrinsic factors related to the GP, patient, and physician-patient relationship were also stated. The most significant conclusion to emerge from this study is that although GPs perceived difficulties and challenges towards multimorbidity, they also have the tools to deal with them: the fundamental characteristics of family medicine. Also, the complex care required by multimorbid patients needs adequate consultation time, multidisciplinary teamwork, and more education/training.

## 1. Introduction

In recent years, there has been a worldwide increasing interest in multimorbidity [1], and this is understandable because of its high prevalence [2] and negative consequences, as multimorbidity is ultimately responsible for 63% of all deaths worldwide [3]. Multimorbidity, the presence of multiple chronic conditions or diseases in the same individual [4,5,6], is becoming progressively more common [7]. Currently, an estimated 50 million people in the European Union suffer from multimorbidity [8], making it the most common chronic condition [5]. Also, in America, the number of people with chronic conditions is projected to increase steadily for the next 30 years [9]. In a recent study in Portugal the prevalence of multimorbidity in primary care was above 70% in adult patients [10].

Multimorbid patients have a higher number of primary care consultations and health-related costs [11]. This has significant implications for the healthcare system and patients’ quality of life [12,13]. Multimorbidity is thus a major challenge to primary care [14]. Nonetheless, general practitioners (GPs), practising closely to the community, are highly-trained to provide appropriate and cost-effective care for patients across their life span [14,15].

Evidently, primary care will play a significant role in future strategies to deal with multimorbidity. For the development of interventions for improving outcomes in multimorbid patients, it is important to assess GPs’ experiences and opinions regarding multimorbidity and its management [16,17].

To date, qualitative studies that have explored the lived experiences of GPs did not find a single unifying result [18]. This may be the consequence of different research methods and distinct health care systems studied. Consequently, interventions towards multimorbidity in primary care in Portugal will have to take into account the country’s own health care particularities, which are known to local practising GPs. Most of the Portuguese population has health care coverage [19] and the primary care centre is commonly the first point of contact with the public system [20]. GPs in primary care centres provide the following services: “general medical care for the adult population; prenatal care; children’s care; women’s health; family planning and perinatal care; first aid; certification of incapacity to work; home visits; preventive services, including immunization and screening for breast and cervical cancer and other preventable diseases” [20] (p. 100). GPs also act as gatekeepers, and the referrals to secondary care are made through them [20].

Portuguese GPs’ views and attitudes will be used to inform health care policy and potential interventions and will also add to the existing international knowledge regarding multimorbidity in other National Health Services with a gatekeeping system in place.

The main aim of this study was to access GPs’ knowledge, awareness, and practices regarding multimorbidity and its management. The second objective was to evaluate the clarity and usefulness of the European General Practice Research Network (EGPRN) definition of multimorbidity [21], recently translated to Portuguese [22]. This is a comprehensive concept of multimorbidity [23] that may have a positive contribute for a future consensual definition. A consensus will be important for the comparability of results across studies. The third objective was to study if providing informational material depicting results of our previous studies on multimorbidity, would change current GPs’ views on the subject.

## 2. Materials and Methods

A web-based qualitative descriptive study [24] was carried out in the first trimester of 2016 and represents the third and final phase of the MM-PT project (Multimorbidity in primary care in Portugal) [25]. In general, this project explores the epidemiology of multimorbidity in Portugal [25].

An internet-based approach was employed since it can be an effective alternative to postal and telephone surveys of health professionals [26]. Qualitative data collected by this process has comparable quality to other collection methods, at lower costs and with shorter response times [27].

The current study was conducted in agreement with the principles of the Declaration of Helsinki [28]. Ethical clearance was obtained from an Ethics Committee at Faculty of Health Sciences (University of Beira Interior) and at Central Regional Health Administration (Portugal). The reporting of this study conforms to the Standards for Reporting Qualitative Research (SRQR) guidelines [29].

### 2.1. Sample and Recruitment

Primary care physicians working in two districts of the centre region of Portugal (Coimbra and Aveiro) participated in the study. These districts were conveniently chosen to maximize sample variation since features of the Primary Care centres located in these regions were known to the research team.

Considering that there is no universally established sample size for qualitative research [24], no formal calculations were performed to estimate sample size. Nonetheless, at least 10% of the population of GPs in these two districts, corresponding to a sample size of approximately 60 GPs, was anticipated to be included in the study. Purposive sampling [30] was used in the study with the goal to maximize variation in regard to primary care physicians’ sex, age, academic degree, career level, experience in primary care, and practice type.

Different recruitment strategies were used: the questionnaire’s web address was publicized on medical open web sites and electronic discussion groups and also distributed by chain referral [31] between peers. Monthly reminders were sent. Participation was voluntary and no reimbursement was offered. All respondent GPs have been included and no exclusion criteria was used. Sampling ceased after saturation [32] (i.e., once the research team considered that there was a sufficient variation in respondent characteristics and that a broad range of opinions towards knowledge, awareness, and practices regarding multimorbidity were expressed).

### 2.2. Data Collection

**Figure 1:**
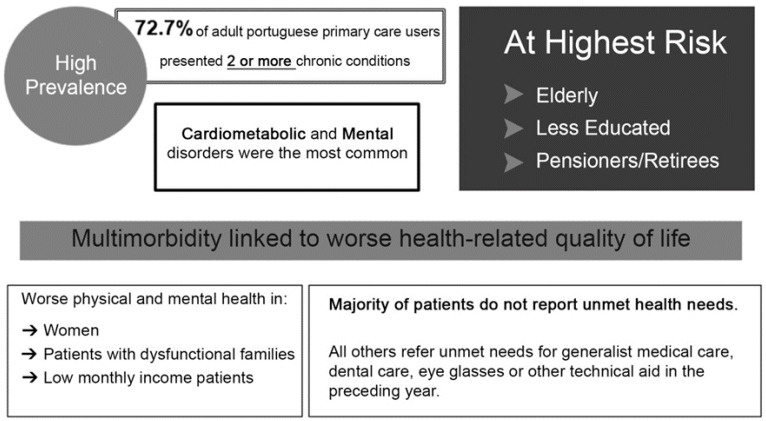
Informational material depicting results of our previous studies on multimorbidity in Portugal.

A questionnaire divided into three sections was designed for data collection. The first section consisted of standard questions concerning respondents’ demographic and professional background information. The second section elicited primary care physician ideas regarding multimorbidity—knowledge (definition of multimorbidity), awareness (relevance of multimorbidity in daily practice), and practices (management of multimorbidity). These main topics were evaluated by the following questions: (1) “Are you familiar with the concept of multimorbidity?” (yes/no); (2) “In your opinion, what is the meaning of multimorbidity?” (open-ended); (3) “How clear is the European General Practice Research Network (EGPRN) concept of multimorbidity?” (extremely/very/moderately/slightly/not at all) (N.B. the Portuguese translation [22] was provided to participants immediately before this question); (4) “How useful is the EGPRN concept of multimorbidity (Portuguese translation) [22]?” (extremely/very/moderately/slightly/not at all); (5) “Give your comments, ideas or suggestions regarding the previously presented definition of multimorbidity” (open-ended); (6) “In your opinion, what is the importance of multimorbidity in your day as a GP?” (open-ended); (7) “In clinical practice, what are the difficulties and challenges that you find in the consultations with patients with multimorbidity?” (open-ended); (8) “In clinical practice, how do you manage the difficulties and challenges found in consultations with patients with multimorbidity?” (open-ended). The third section briefly itemized the available results from the previous phases of the MM-PT project [10,25,33] (Figure 1) and questioned the respondents if after reading the information provided they would change their former ideas regarding the (1) concept of multimorbidity (no/yes; justify your choice: open-ended); (2) importance of multimorbidity (open-ended); (3) primary care physicians’ clinical practice (open-ended). The last section also allowed respondents to manifest comments, ideas, or suggestions regarding the MM-PT project’s results.

The questionnaire was posted online after being pre-tested and reviewed by a panel of experts in multimorbidity and experienced GPs in order to check its comprehensibility. The questionnaire was completed anonymously. Mean response time was 15 min. Incomplete questionnaires were not included in the analysis.

### 2.3. Data Analysis

Open-ended questions were analysed via inductive thematic content analysis [34,35]. This process followed the recommendations of Braun and Clarke [35]. No computer-assisted qualitative data analysis software was used, since open-ended data analysis are commonly done by human coding [36]. In brief, the study investigator tagged (by using code names) the segments of text that described distinctive ideas. Similar codes were grouped together to delineate themes. This procedure was revised by an independent expert and results were further discussed until a consensus was achieved. The concepts and categories that emerged from the Portuguese qualitative data were translated to English as described by Chen and Boore [37].

Basic descriptive statistics from questionnaire data were done using the IBM SPSS Statistics for Windows, Version 21.0 (IBM Corporation, Armonk, NY, USA).

## 3. Results

Seventy-four (51 females and 23 males) of 122 primary care physicians completed the questionnaire. Table 1 summarizes respondents’ characteristics. There was sufficient variation in sex, age, academic degree, career level, experience in primary care, and practice type.

### 3.1. Definition of Multimorbidity

The majority of the respondents (62/74) were familiar with the concept of “multimorbidity”. Nonetheless, its definition varied within the sample.

Almost all respondents (68/74) considered multimorbidity as having multiple diseases (or health problems), whereas a few suggested it to be equal to multipathology or polypathology (Quote 1) (see also Quote 3). Twenty-four respondents considered only chronic diseases and four both chronic and acute diseases in their own definitions.

“*Several diseases coexist in the same patient, particularly chronic and of complex clinical management, which may interfere with his quality of life, autonomy, and ultimately longevity*.”

(Quote 1 Respondent 39)

Cut-off counts of two, three, and five chronic diseases were suggested by the respondents (23/74). The cut-off of two chronic diseases was the most frequently referred (19/74).

Some definitions were more complex. They included some negative outcomes of multimorbidity and its management challenges (Quote 1, Quote 2).

“*Presence of two or more chronic diseases in the same person causing decreased quality of life, increased demand for health resources and also creating challenges in patients’ treatment and counselling*.”

(Quote 2 Respondent 8)

One respondent (Quote 3) used the term “health problem” since it can be more inclusive in the primary care context than the term disease which is characterized by specific signs and symptoms.

“[…] *means having several pathologies, or in this case, health problems. Partly it is synonymous to multiple pathologies, but in the context of Primary Health Care, goes further than that, because not all health problems are actually diseases*.”

(Quote 3 Respondent 43)

Another respondent (Quote 4) referred the lack of an index disease when defining multimorbidity.

“*Unlike comorbidity this concept [multimorbidity] does not place a disease as central and others as satellites. All have a contributing role*.”

(Quote 4 Respondent 74)

#### Definition of Multimorbidity by the European General Practice Research Network (EGPRN)

The majority of the respondents (50/74) considered the EGPRN’s definition of multimorbidity to be very/extremely clear. A slight smaller proportion (40/74) found it to be very/extremely useful for primary care.

“*It is very important to better identify patients with multimorbidity. It is very complete. I agree with this definition*.”

(Quote 5 Respondent 52)

For only a select few this definition has limited use for primary care since it can be too complex, extensive, and its various subterms (such as “biopsychosocial factor” and “somatic risk factor”) are lacking operationalization.

### 3.2. Relevance of Multimorbidity in Daily Practice

All the respondents made comments endorsing the importance of multimorbidity in everyday practice (74/74). They recognize that multimorbidity is “inextricably linked to general practice” because multimorbid patients have a high prevalence in primary care settings and single disease patients are the exception (25/74).

“*It is very prevalent. In an aging population, there is a large percentage of people who are walking medical textbooks [have every illness you can imagine]. Stress factors, unemployment, poor working conditions, the presence of a dependent elderly [in the household], diseases in family member etc. are factors that aggravate this situation, I believe that single disease patients have no expression in my daily practice*.”

(Quote 6 Respondent 7)

In addition to the stated epidemiologic theme, other respondents went further and referred that the importance of multimorbidity in primary care is attributable to the difficulties and challenges of managing multimorbid patients. This is further described below.

### 3.3. Perceived Difficulties and Challenges

Two broad themes emerged from the analysis. Difficulties and challenges perceived by the respondents were felt at a systemic level, regarding the Heath Care System, and at an individual level, regarding the general practitioner and the patient (Table 2).

#### 3.3.1. Difficulties and Challenges Inherent to the Health Care System

##### *Lack of resources:* 

Respondents’ most important lacking resource was consultation time. They stated that the Portuguese Health Care System’s “consultation time is short”, insufficient to “listen to the patient and his multiple complaints” and hinders the GP’s assessment of the entire perspective on the patient’s situation thereby resulting in inappropriate, fragmented care (Quote 7).

“*Lack of time to be able to see the big picture, ending always to work smaller parts at a time and the results are not always good, it leads to forgetfulness, [treatment] redundancies, delays [in diagnosis]...*”

(Quote 7 Respondent 17)

The shortage of multidisciplinary healthcare professionals (e.g., psychologists, nutritionists, dentists, etc.) was also mentioned as one of the reasons leading to excessive demand for the use of primary care services and increased amount of work for the GP.

Respondents also stated that the limited and unadjusted information and communication technologies obstruct retrieval and transfer of important medical data and do not provide drug information regarding contraindications and interactions, thus interfering with patient care. 

##### *Organisational barriers between primary and secondary care providers:* 

Respondents highlighted the current lack of collaboration between secondary and primary care providers. This was felt at several levels: (1) accessibility—lack of timely appointments in secondary care; (2) communication—inefficient feedback from secondary care providers; (3) secondary care provider role—absence of coordinated care (Quote 8).

“*Secondary care providers do not deliver a global care [for the multimorbid patient], but fragmented [focusing on a specific health problem], because there is no hospital physician (e.g. internist), in straight connection with the GP, to act as a care manager for these patients*”

(Quote 8 Respondent 10)

#### 3.3.2. Difficulties and Challenges External to the Health Care System

##### *Media pressure:* 

Participants mentioned that the pressure from the media is a barrier to patient care. Although these statements were very generic, and did not provide more details on how this pressure is manifested (e.g., “we feel pressured by the media when treating our patients”).

##### *Insufficient patient support:* 

Respondents recognized that the present unavailability of resources that could be provided by community-based support services and/or by family members increases workload for the GP and makes GPs responsible for everything regarding the care of their patients (Quote 9).

“*The lack of support [...] to help solve many of the problems (which are not organic diseases) that affect the physical well-being of multimorbid patients creates an excessive demand for the use of primary care services. The GP feels powerless to solve social, work and family related problems*.”

(Quote 9 Respondent 52)

#### 3.3.3. Difficulties and Challenges Related to the GP

##### *GP’s role of treating the whole person:* 

Participants stated that the GP’s role of providing a whole person health care to multimorbid patients is challenging (Quote 10).

“*Managing multimorbidity is hard work for GPs because we focus on the health of the whole person. And the whole person is difficult to manage pharmacological and non-pharmacologically*.”

(Quote 10 Respondent 48)

Several reasons were referred. Respondents considered that it is demanding to make a holistic assessment of the multimorbid patient because of the difficulties of obtaining an accurate history from elderly patients and with low educational levels. Consequently, it becomes challenging to negotiate priorities and goals tailored to the patient agenda. Respondents also refer that they feel pressured to follow clinical indicators/guidelines and ultimately they experience emotional distress with feelings of inability to help.

##### *Medical education:* 

Participants stated that they have “insufficient training and practice in the topic of multimorbidity”. They also mentioned that it is “difficult to try to keep up to date with medical knowledge since the multimorbid patient can suffer from countless conditions at the same time”.

#### 3.3.4. Difficulties and Challenges Related to the Multimorbid Patient

##### *Diagnostic challenges and complex clinical management:* 

Respondents pointed out diagnostic and therapeutic challenges when dealing with multimorbid patients. Clinical cases are more complex and difficult to handle since their conditions may be masked by multiple overlapping symptoms. Polypharmacy was extensively mentioned (Quote 11) as the most common therapeutic challenge in multimorbidity. Due to the need to treat numerous conditions and since guidelines are single-disease oriented, these will increase the use of multiple drugs per patient with an increased risk of iatrogenesis (effects of possible drug-disease and drug-drug interactions and medical error) and also of low levels of medication adherence.

“*Two common areas of difficulty are polypharmacy and health promotion, since taking into account what is best for a condition may worsen another*.”

(Quote 11 Respondent 8)

Participants mentioned that they also have difficulties in recognising what conditions and outcomes are most important for the patient and for the GP, how to avoid treatments that lack solid supporting evidence, and how to deprescribe.

##### *Poor patient engagement:* 

Respondents noted that multimorbid patients are poorly engaged in their own treatment. They have limited health literacy skills, do not acknowledge the future implications of multimorbidity, do not comprehend the health-related information communicated by the GPs, and do not adopt healthier lifestyles because of their belief in personal invulnerability.

**Figure 2:**
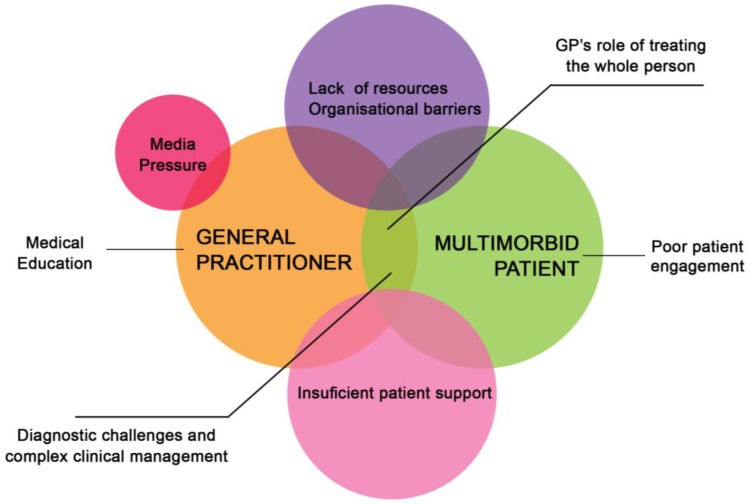
Relations between the perceived difficulties and challenges.

The possible relations between the different sets of perceived difficulties and challenges were considered in the diagram depicted in Figure 2.

### 3.4. Management of Multimorbidity

Analysis of the data revealed seven main themes (Table 3).

Participants stated that they are fully committed to helping their patients with multimorbidity even though they find it to be a very difficult task and a source of distress.

“…*with great difficulty and distress due to the [short] consultation times, allied with constant interruptions by various coworkers, patients are often inevitably cut short in the exposure of their concerns. Owing to the lack of existing human resources, family doctors are then required to see to not only their list of patients as well as others whose doctors are absent and in need of urgent care. This situation is not easy to solve*.”

(Quote 12 Respondent 53)

Characteristics commonly associated to family medicine [15] were mentioned by the respondents as the tools used in daily practice to manage the difficulties and challenges of multimorbidity: (1) person centeredness—“focus care on the person and not on diseases”, “know the patient, his background (myths and expectations), living situation and family dynamics”, “reconcile doctor and patient agendas” by being “aware of each patient’s needs and priorities”, “promote patient empowerment by educating and keeping them and their families well informed” and “get patients to take responsibility for their own health”; (2) holistic model—“make a global approach, never forgetting the dimensions (bio-psycho-social) of the patient”; (3) effective doctor-patient relationship —take advantage of “empathy”, “proximity”, “patience”, and “perseverance” in the therapeutic relationship, “use clear and straightforward language in the doctor-patient communication”; (4) integrated approach—“disease prevention efforts” should be devised as well as a focus on managing multiple conditions; (5) continuing management—“offer longer consultations” (including online doctor consultations), “increase the number of consultations”, and arrange a “short span of time between consultations”; (6) coordination with others and teamwork—“work together with other health care professionals (generally with the nurses)”, “include inter-organisational collaboration”, and “cooperate with families and other carers”; (7) problem solving skills—“attend continuing education courses and postgraduate educational activities”, “balance the best available evidence with the experience based medicine”, “optimize drug prescribing by avoiding the tendency to medicalize, negotiating treatment with the patient, updating the patient’s medication list at each visit, deprescribing when needed, and by using non drug therapies”.

### 3.5. Informational Material Bearing the Results from Previous Portuguese Multimorbidity Studies

After reading the informational material provided (Figure 1), (1) approximately one-quarter of the sample (18/74) would amend their previous definition of multimorbidity. The most frequently stated change was the inclusion of “social factors” and the “negative influence of multimorbidity on quality of life” in the definition (one respondent would add the “need of multidisciplinary care” and another participant thought that it was important to complement the definition with “difficulty in access to care when needed”); (2) nearly the entire sample highlights the extremely high importance of multimorbidity—“data shown accentuates the importance of multimorbidity and the need to establish strategies for dealing with this condition, particularly among the elderly”, “it is much more prevalent than sometimes we remember”, “the maximum importance, underlining the psychosocial aspects”, “extremely important as it can be a generator of burn-out among health professionals”, “it is a serious problem for the (Portuguese) National Health Service”; (3) the majority (52/74) will change their daily practice regarding multimorbidity—“even more dedication to the diagnose and management of patients with multimorbidity”, “further improve accessibility for the at risk and vulnerable groups”, “pay more attention to the management of the most at risk groups of multimorbidity and take into special consideration the mental and cardiometabolic illnesses”, “start applying instruments of measurement of quality of life”, “training in the management of the most prevalent diseases and the interactions of different drugs used in their treatment”, “create a distinct consultation for multimorbid patients”, “give at least 30 min consultations for these patients”.

## 4. Discussion

The current study found high levels of awareness regarding multimorbidity within its participants. In accordance with available literature [21,38,39], no universally accepted definition of multimorbidity was found, and the concept was heterogeneous between respondents [39]. Interestingly, none of the definitions were incorrect. This highlights the complexity of this area of research and also the importance of finding a consensus on how multimorbidity is defined. 

When queried about the EGPRN’s definition of multimorbidity (Portuguese translation [22]), the sample recognized the clarity and usefulness of the definition for primary care settings. This result may be explained by the fact that EGPRN’s definition is comprehensive [21], more adapted to the complexity of the multimorbid patient [23], and eventually superior for clinical purposes than the commonly used definition of co-occurrence of two or more long-term conditions in the same patient [40].

This study adds to findings from previous studies of GPs’ views and attitudes in multimorbidity [16,17,18,41,42,43,44]. To our knowledge, this is the first study of its kind done in Portugal. Our sample included sufficient variation in sex, age, academic degree, career level, experience in primary care, and practice type, which provided a deeper understanding of GPs’ subjective perceptions. All respondents were practising physicians and therefore provided real-world data.

A shared view amongst respondents was that multimorbidity is very common and associated with old age, which supports former qualitative research reporting GPs’ perspectives [41] and is consistent with data obtained from epidemiologic studies [10]. 

GPs pointed out several difficulties and challenges while managing multimorbidity. As expected, common consequences of these drawbacks are a significant burden related to patient management and the toll on patient care [16,41,43].

Perceived difficulties and challenges could be classified on the basis of their relation to the GP or the patient into two types, extrinsic and intrinsic.

Extrinsic factors were associated with the healthcare system logistics management (consultation time, organization of care teams, clinical information) and society (media pressure, social/family support). These practical issues seem to be consistent with the ones identified in earlier studies [43,44], with the exception of the “media pressure” topic that has not previously been reported in multimorbidity. Partial media coverage may have a negative impact on patient care [45] and although austerity measures are associated with increased mortality [46], in the last few months Portugal’s healthcare system was targeted by the media concerning cuts in the health service and mortality cases [47], which consequently may put pressure on physicians in general and particularly on GPs that manage complex multimorbid patients. This will certainly require further study.

The perceived extrinsic factors demonstrate the necessity for longer consultations [18,41,42,48]. In Portugal, the average consultation length in general practice is approximately 15 min [49,50], similar to Belgium and Switzerland, and longer than Germany, Spain, the Netherlands, and the United Kingdom [51]. Usually GPs do not have enough time to manage patients with chronic diseases [52], but when they do, it decreases GPs stress and increases patient enablement [53]. If impossible, GPs may adopt time-management strategies [54] and take advantage of efficient health information technologies [55] to warrant more effective consultations. There is also the need for team-based care [56] that includes other co-workers in addition to the GPs (e.g., psychologists, nutritionists, dentists, care coordinators, etc.), cooperation with families and social organizations for better patients’ social support [43,44], and improvement of referral systems for hospital care [43]. In Portugal, there is a known lack of coordination between specialist care and primary care with a large number of patients bypassing their GP by visiting emergency departments [20]. The referral rate from primary to secondary care is approximately 6% [57,58], which is similar to the situation in Spain [59]. The waiting times for specialist care may vary widely from one to six months [60], and feedback from secondary care providers is received in less than 40% of the cases [58,60,61].

Intrinsic factors related to the GP, patient, and physician-patient relationship were also stated. In the recent review of Cottrell and Yardley [18] and in the present study, GPs acknowledged the complexity in managing multimorbidity with an increasing workload [41,44,62]. GPs faced difficulties and challenges in delivering holistic care [16], they experienced feelings of inability to help considering existing resources, and stated lacking competences in dealing with multimorbidity [41,43,44], including uncertainty on how to recognise what conditions and outcomes are most important for the patient and for the GP, how to avoid treatments that lack solid supporting evidence, and how to deprescribe. Inadequacy of guidelines and polypharmacy were also mentioned as major therapeutic challenges, as shown in previous studies [16,44]. Difficulties in communicating with multimorbid patients, frequently elderly and individuals with low education levels, may be the reason of poor patient engagement. Some researchers have emphasized that physicians with better communication and interpersonal skills are able to perform more quality consultations [63].

Characteristics fundamental to family medicine [15] were mentioned by the respondents in an extremely positive and optimistic way as the tools that could be used in daily practice to manage the challenges of multimorbidity. The current results match those of Le Reste et al. [23], which indicated that GPs consider these characteristics as a valid contribute to the detection and management of multimorbid patients [23]. In the study of Luijks et al. [17] and in the present study, a person-centred approach was considered to be the crucial intervention strategy for multimorbidity. A key element of such an approach in family medicine is the “understanding of the patient as well as his disease” [64] (p. 24). Some researchers have highlighted the value of individualised care not only for GPs but also from the patients’ perspectives [18], including a better physician-patient relationship [65].

The informational material provided concerning data on multimorbidity in Portugal was able to increase consciousness regarding the importance of multimorbidity and at the same time was capable of driving change in the way GPs deal with multimorbidity and multimorbid patients in their daily practice. This material was well received by the GPs. One respondent, a GP 62 years of age, made the following final comment: “There should be more studies like these. Researchers should whenever possible disseminate the results of their previous studies and ask for opinions as did this colleague of ours. Thank you and congratulations.” Providing short informational materials to GPs may also be one way to bring together clinical research and clinical practice, which in turn benefits patients and healthcare as a whole [66].

The main limitations in this study are similar to the ones presented in previous qualitative studies regarding GPs’ perceptions of multimorbidity. Although not the objective of the study, current data does not directly evaluate GPs’ daily practices but only what they perceive they do [16]. Future research with a different design should be undertaken to investigate this further. Patient views and also their caregivers were not sought in the current study and will require consideration in following research [16] in Portugal.

## 5. Conclusions

One of the more significant findings to emerge from this study is that although GPs are overwhelmed by the difficulties and challenges of multimorbidity, at the same time, they have the tools to deal with them: the fundamental characteristics of family medicine. Also, the complex care required by multimorbid patients needs adequate consultation time, multidisciplinary teamwork, and more education/training. Improvements to the organization of care delivery are mandatory and this study provided data that can be used to plan future interventions towards multimorbidity in primary care in Portugal.

## Figures and Tables

**Table 1:** Physicians characteristics (*n* = 74).

Characteristic	*n* (%)	Mean (SD)
**Sex**		
Women	51 (68.92)	
Men	23 (31.08)	
**Age (years)**		43.73 (13.78) min = 26; max = 64
**Academic degree**		
Entry-level medical degree (MD)	55 (74.32)	
Higher medical degrees (Postgraduate/Master/PhD)	19 (25.68)	
**Career level**		
General practitioner (GP)	54 (72.97)	
GP in training	20 (27.03)	
**Experience in primary care (years)**		16.19 (13.29) min = 1; max = 37
**Practice type**		
Family Health Unit (family practice based model)	50 (67.57)	
Personalized Healthcare Unit (individual based model)	24 (32.43)	
**Place of work (district)**		
Coimbra	35 (47.30)	
Aveiro	39 (52.70)	

**Table 2:** Difficulties and challenges.

**Systemic Level: Health Care System**	Inherent to the Healthcare System	Lack of resources: consultation time restraints; interdisciplinary care/teams; computing and informatics Organisational barriers between primary and secondary care providers
	External to the Healthcare System	Media pressure Insufficient patient support: community-based support services, family support
**Individual Level: General Practitioner and Multimorbid Patient**	General Practitioner related	GPs role of treating the whole person: reconciling doctor-patient agenda; doctor-patient communication difficulties; feelings of inability to help; pressure to follow clinical indicators/guidelines Medical education
	Multimorbid Patient related	Diagnostic challenges and complex clinical management Poor patient engagement

**Table 3:** Tools used by GPs to manage the difficulties and challenges of multimorbidity.

(1) person centeredness
(2) holistic model
(3) effective doctor-patient relationship
(4) integrated approach
(5) continuing management
(6) coordination with others and teamwork
(7) problem solving skills
